# Long non-coding RNAs regulate drug resistance in cancer

**DOI:** 10.1186/s12943-020-01162-0

**Published:** 2020-03-12

**Authors:** Kaisheng Liu, Lin Gao, Xiaoshi Ma, Juan-Juan Huang, Juan Chen, Leli Zeng, Charles R. Ashby, Chang Zou, Zhe-Sheng Chen

**Affiliations:** 1 The First Affiliated Hospital of Southern University of Science and Technology, The Second Clinical Medical College of Jinan University, Shenzhen People’s Hospital, Shenzhen, 518020 Guangdong People’s Republic of China; 2 Department of Physics, Technical University of Munich, 85748 Garching, Germany; 3 College of Pharmacy and Health Sciences, St. John’s University, Queens, New York, NY 11439 USA; 4 Tomas Lindahl Nobel Laureate Laboratory, Research Centre, The Seventh Affiliated Hospital, Sun Yat-sen University, Shenzhen, 518107 Guangdong People’s Republic of China

**Keywords:** Cancer, Drug resistance, Long non-coding RNAs

## Abstract

Chemoresistance, whether intrinsic or acquired, is a major obstacle in the treatment of cancer. The resistance of cancer cells to chemotherapeutic drugs can result from various mechanisms. Over the last decade, it has been reported that 1ong noncoding RNAs (lncRNAs) can mediate carcinogenesis and drug resistance/sensitivity in cancer cells. This article reviews, in detail, recent studies regarding the roles of lncRNAs in mediating drug resistance.

## Background

Globally, cancer is the leading cause of mortality and in 2018, it was estimated that there were 9.6 million cancer-related deaths [1]. Currently, the primary therapeutic approaches for treating cancer are chemotherapy, radiation and surgery [2]. However, during treatment, tumor cells can become resistant to chemotherapy due to, but not limited to; 1) increased expression of certain ATP-binding cassette (ABC) transport proteins that decrease the intracellular concentration of anticancer drugs, thereby decreasing their efficacy; 2) alterations that allow cancer cells to avoid cell death; 3) increase in DNA repair; 4) mutations in specific cellular targets; 5) alterations that allow cancer cells to tolerate adverse or stressful conditions and 6) increasing the biotransformation of anticancer drugs to less efficacious or inactive metabolites [3]. Consequently, drug resistance is still a major challenge as it often causes therapeutic failure [4]. Furthermore, drug resistance can be present in tumor cells before chemotherapy, a phenomenon known as acquired drug resistance [5]. Overall, the underlying mechanisms of resistance to chemotherapeutic drugs remain to be fully elucidated.

The development of new technologies, in combination with bioinformatics, has resulted in the discovery of additional genes associated with drug resistance [6]. Furthermore, it is important to note that < 2% of the human genome encodes proteins and 98% of the transcriptional products are short and long non-coding RNAs (lncRNAs) [7, 8]. LncRNAs consist of more than 200 nucleotides and have no protein coding function [7]. LncRNAs are less conserved among species, are typically expressed at low levels and often have high tissue and development specificity [9]. LncRNAs have important regulatory roles in many aspects of genome function, including gene transcription, splicing, and epigenetics, as well as biological processes involved in the cell cycle, cell differentiation, development, and pluripotency [10]. LncRNAs have recently been identified as a new mechanism in drug resistance/sensitivity and has garnered significant attention in the area of cancer research. Indeed, numerous papers have been published over the last decade regarding lncRNA and resistance to anticancer drugs. In this review, we will discuss the mechanisms by which lncRNAs produce drug resistance in cancer cells.

### LncRNA roles in mediating resistance to anticancer drugs

#### The effect of lncRNA on phase I and phase II enzymes

Alterations in drug metabolism are one of the important and most studied mechanisms that mediate drug resistance. The mechanisms of drug metabolism and disposition can be categorized as: phase I, phase II and phase III [11]. LncRNAs can regulate certain phase I enzymes and affect drug resistance in cancer cells. For example, lncRNA H19 is overexpressed in colorectal cancer and increases the intracellular aldehyde dehydrogenase (ALDH) activity in colorectal tumors [12]. LncRNA H19 activates the β-catenin pathway by sequestering miR-141, which contributes to tumor development and chemoresistance in colorectal cancer tumors [12].

LncRNAs have been shown to affect the regulation of specific phase II enzymes [13, 14]. The expression of the lncRNA, HOX transcript antisense intergenic RNA (HOTAIR), is positively correlated with the level of the carbohydrate sulfotransferase (CHST15) protein in primary, as well as the number of metastatic tumor lesions [13]. In addition, HOTAIR promotes the invasion of breast cancer cells by affecting the expression of cell surface glycosaminoglycans [13]. The lncRNA *Homo sapiens* glutathione S-transferase mu 3, transcript variant 2 and noncoding RNA (GSTM3TV2) levels are significantly increased in pancreatic tumor tissues and it upregulates the L-type amino acid transporter 2 (LAT2) and oxidized low-density lipoprotein receptor 1(OLR1) by competitively sequestering let-7 (a mRNA targeting c-Myc, HMGA2 and Ras) to induce gemcitabine resistance in pancreatic cancer [15].

#### Altered drug efflux and related lncRNAs

**Fig. 1:**
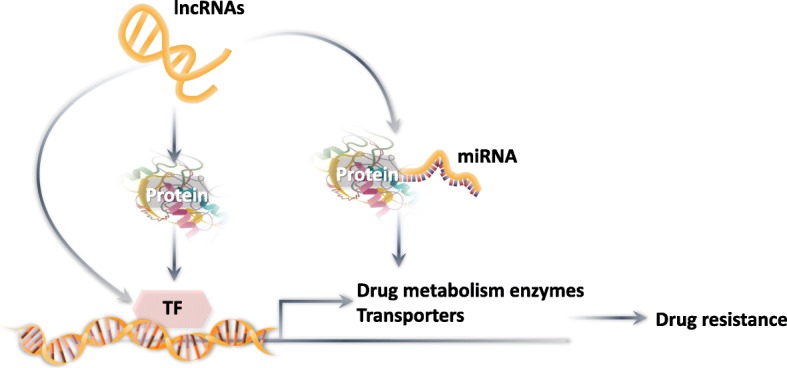
Schematic illustration of lncRNA-induced resistance to anticancer drugs by altering drug metabolism and drug efflux

In phase III drug disposition, the metabolites of the drugs are eliminated and excreted by various endogenous transporters that are found in the liver, small intestine, brain and kidney, which play a role in protecting tissues and organs from endogenous and xenobiotics [16, 17]. It is well established that the overexpression of the ABC proteins by cancer cells, which efflux anticancer drugs from the cancer cells, thereby attenuating or abrogating their efficacy, mediates resistance to certain anticancer drugs [18, 19]. Numerous studies indicate that the members of the ABC transporter family associated with multidrug resistance (MDR) in cancer cells include p-glycoprotein (P-gp/ABCB1), MRP1/ABCC1, MRP2/ABCC2, MRP4/ABCC4, and BCRP/ABCG2 [20] (Fig. 1).

Recent studies have shown that specific lncRNAs can affect various ABC transporters, thereby producing drug resistance. For example, in hepatocellular cancer (HCC), knockdown of lncRNA H19 significantly increased the methylation of the MDR1 promoter methylation and decreased MDR1/P-glycoprotein expression in doxorubicin (DOX)-resistant R-HepG2 cells [21]. In addition, the levels of lncRNA very low density lipoprotein receptor (VLDLR) are significantly increased in HCC, and the knockdown of lncRNA VLDLR significantly reduced HCC proliferation and the expression of BCRP/ABCG2, while overexpression of BCRP/ABCG2 decreased the effect of lncRNA VLDLR1 knockdown on sorafenib-induced cell death in HepG2 cells [22]. The lncRNA plasmacytoma variant translocation 1 (PVT1) is highly expressed in gastric cancer tissues of cisplatin-resistant patients and cisplatin-resistant cells [23]. The up-regulation of lncRNA PVT1 increased the expression of MDR1, MRP, mammalian target of rapamycin (mTOR) and hypoxia-inducible factor alpha (HIF-1α) and decreased the apoptosis produced by cisplatin in BGC823 and SGC7901 cancer cells [23]. The lncRNA MDR-related and upregulated lncRNA (MRUL) was significantly upregulated in the multidrug-resistant gastric cancer cell sublines, SGC7901/ADR [resistant to doxorubicin/adriamycin (DOX/ADR)] and SGC7901/VCR [resistant to vincristine (VCR)], and its expression significantly decreased the anti-proliferative efficacy of ADR or VCR [24]. The expression of lncRNA MRUL increases the expression of P-gp/ABCB1 in an orientation- and position-independent manner and the depletion of MRUL decreased ABCB1 mRNA levels in a concentration - and time-dependent manner [24]. In addition, the knockdown of lncRNA AK022798 downregulated the expression of MRP1/ABCC1 and P-gp/ABCB1, and increased apoptosis and the expression of caspase - 3 and caspase - 8 in the cisplatin-resistant gastric cancer cell lines, SGC7901/DDP and BGC823/DDP [25]. The lncRNA metastasis-associated lung adenocarcinoma transcript 1 (MALAT1) significantly upregulates MRP1/ABCC1 and MDR1/ABCB1 by activating STAT3 in a cisplatin (DDP) resistant non-small cell lung cancer cells [26]. The lncRNA antisense non-coding RNA in the INK4 locus (ANRIL) was highly expressed in the gastric cancer tissues of cisplatin-resistant and 5-fluorouracil (5-FU)-resistant patients, and in cisplatin-resistant cells (BGC823/DDP) and 5-FU-resistant cells (BGC823/5-FU) [27]. The knockdown of the lncRNA ANRIL decreased the expression of MDR1/ABCB1 and MRP1/ABCC1, and increased the efficacy of cisplatin or 5-FU in the cisplatin-resistant cell line, BGC823/DDP or the 5-FU-resistant cells, BGC823/5-FU [27]. The lncRNA KCNQ1OT1 is highly expressed in lung adenocarcinoma cells and the knockdown of lncRNA KCNQ1OT1 significantly decreased the expression of MDR1/ABCB1 in A549 adenocarcinomic human alveolar basal epithelial/human ovary cells derived from metastatic site (PA1) cells [28]. The knockdown of lncRNA X-inactive specific transcript (XIST) upregulates miR-124 and downregulates serum and glucocorticoid-inducible kinase 1 (SGK1), which increases the in vivo efficacy of DOX in colorectal cancer cells by facilitating DOX-induced apoptosis [29]. The expression of both lncRNA linc00518 and MRP1/ABCC1 are significantly increased in human breast cancer tissues compared to normal adjacent tissues [30]. The downregulation of lncRNA linc00518 increased the efficacy of DOX, vincristine and paclitaxel in MCF-7 breast cancer cells resistant to ADR and increased the anti-tumor efficacy of ADR in vivo by regulating miR-199a/MRP1 axis in MCF-7/ADR cells [30]. Finally, the lncRNA bladder cancer associated transcript-1 (BLACAT1) decreases the efficacy of oxaliplatin, a P-gp/ABCB1 substrate, by increasing the expression of the ABCB1 protein via sponging miR-361, which targets 3′-UTR of BLACAT1 and ABCB1 mRNA [31].

#### The inhibition of apoptosis by lncRNAs

**Fig. 2:**
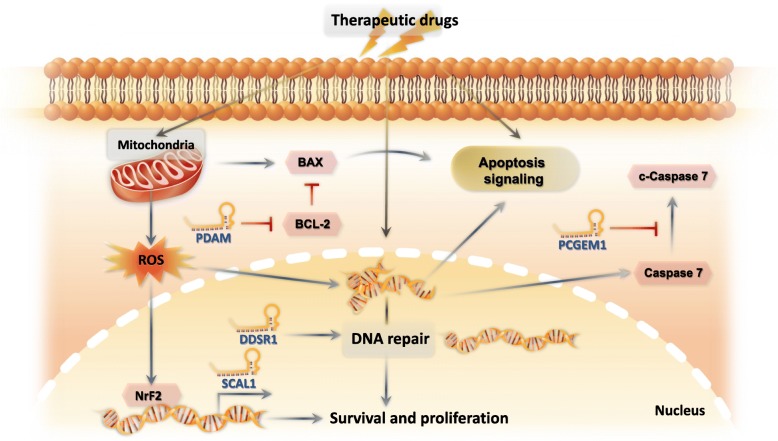
LncRNAs protect cells from anti-cancer drugs by suppressing apoptosis caused by oxidative stress or DNA damage. The lncRNA SCAL1 protects cancer cells from chemotherapeutic drug-induced oxidative damage by upregulating the transcription factor, NrF2. The lncRNA DDSR1 produces cisplatin resistance by increasing DNA repair. The lncRNA PCGEM1 prevents apoptosis by inhibiting the activation of caspase - 7. The loss of lncRNA PDAM inhibits cisplatin induced-apoptosis by upregulating the expression of the anti-apoptotic protein, BCL-2

Numerous studies have shown that the majority of chemotherapeutic drugs used in the treatment of cancer induce cell death by activating apoptosis pathways and the dysregulation of apoptosis produces drug resistance and enhance the survival of cancer cells [32, 33]. Recently, the expression levels of lncRNAs have been reported to be significantly correlated with drug resistance in various tumors. The lncRNA E2F1-regulated inhibitor of cell death (ERIC) is a 1.7 kb transcript up-regulated by E2F1 [34]. The knockdown of ERIC significantly increases etoposide-induced apoptosis in osteosarcoma cells incubated with etoposide, suggesting that ERIC plays a role in mediating etoposide resistance [34]. The loss of lncRNA p53-dependent apoptosis modulator (PDAM) increased the expression of the anti-apoptotic protein, BCL-2, which induces cisplatin resistance in oligodendroglial tumors (Fig. 2) [35]. The overexpression of the lncRNA prostate cancer gene expression marker 1 (PCGEM1) produces resistance to DOX-induced apoptosis by suppressing the cleavage of caspase - 7 in LNCaP (cancer cells isolated from the lymph node of a patient with prostate cancer) (Fig. 2) [36]. The lncRNA cancer upregulated drug resistant gene (CUDR) is an urothelial cancer associated 1 (UCA1) transcript variant that is upregulated in many types of tumors [37]. The overexpression of CUDR inhibits apoptosis induced by cisplatin and increases tumorigenesis in bladder cancer cells [37]. Moreover, CUDR produces resistance to DOX and etoposide in squamous cell cancer [38].

#### The repair of damaged DNA by lncRNAs

DNA damage, which is produced by ultraviolet radiation, ionizing radiation and genotoxic chemicals, occurs on a constant basis [39]. DNA damage can be repaired or tolerated in normal cells so as to maintain cellular and organ functions [40]. However, recurrent chemoresistant cancer cells activate the DNA damage response more efficiently and have a higher tolerance in genotoxic stress environments produced by chemotherapeutic drugs compared to primary cancer cells [41].

Several lines of evidence indicate that lncRNA expression is significantly altered in drug resistant cancers. Cisplatin, which damages DNA and induces cell death, is a commonly used chemotherapeutic drug for the treatment of non-small cell lung cancer (NSCLC) [42]. The lncRNA DNA damage-sensitive RNA1 (DDSR1), by interacting with BRCA1 and hnRNPUL1, increases DNA repair by inducing homologous recombination, thereby increasing cisplatin resistance in NSCLC (Fig. 2) [43, 44]. Furthermore, a number of p53-regulated lncRNAs are stimulated in response to DNA damage induced by chemotherapeutic drugs. The lncRNA p21 associated ncRNA DNA damage activated (PANDA) is activated by p53 and interacts with the protein, nuclear transcription factor Y subunit alpha (NFYA, one of the subunits of the trimeric protein, NF-Y, that interacts with CCAAT motifs in promoter regions [45]) to inhibit DNA damage-induced apoptosis in FL3 cells incubated with DOX [46]. The lncRNA HOTAIR produces cisplatin resistance in NSCLC by downregulating p21, an inhibitor of cyclin-dependent kinase that causes cell cycle arrest after DNA damage or overexpression of p53 [47, 48].

#### Oxidative stress and lncRNAs

Reactive oxygen species (ROS) are cell signaling molecules produced in mitochondria during normal cell metabolisms and high concentrations of ROS can cause oxidative stress, producing cytotoxicity in certain cellular environments [49–51]. Aberrant or dysregulated ROS-scavenging systems in cancer can decrease the susceptibility to oxidative stress, resulting in drug resistance [52].

LncRNAs play essential roles in the cellular response to oxidative stress. The ncRNA smoke and cancer-associated lncRNA-1 (SCAL1) is up-regulated by the protein transcription factor, nuclear factor erythroid 2-related factor 2 (Nrf2) in different lung cancer cell lines [53]. As Nrf2 plays an important role in protecting normal cells from oxidative stresses and mediating chemoresistance in certain cancer cells, SCAL1’s increased expression in lung cancer cells suggests that it may provide protection from oxidative stress induced by certain chemotherapeutic drugs (Fig. 2) [53, 54]. The lncRNA transient receptor potential cation channel subfamily M member 2 antisense (TRPM2-AS) codes for an oxidative stress-activated ion channel that regulated cell survival [55]. The lncRNA TRPM2-AS is overexpressed in the prostate cancer cell line, PC3, and its knockdown induced PC3 apoptosis and increased the intracellular levels of hydrogen peroxide, a potent oxidative molecule [55]. Furthermore, recent studies indicate that TRPM2 may have a protective effect in cells exposed to moderate oxidative stress [56, 57] (Table 1).

**Table 1:** LncRNAs-induced cell death in drug - resistant cancer cells

LncRNA	Cancer type	Drug resistant	Mechanisms	Ref
ERIC	Osteosarcoma	Etoposide	ERIC inhibits DNA damage-induced apoptosis	[34]
PDAM	Oligodendroglial tumor	Cisplatin	Loss of PDAM inhibits apoptosis by increasing the expression of BCL-2	[35]
PCGEM1	Prostate cancer	DOX	Overexpression of PCGEM1 inhibits apoptosis by suppressing the activation of caspase 7	[36]
CUDR	Bladder cancer	Cisplatin	Overexpression of CUDR suppresses DNA damage-induced apoptosis	[37]
DDSR1	Non-small cell lung cancer	Cisplatin	DDSR1 inhibits DNA damage-induced apoptosis by promoting DNA repair with homologous recombination	[43]
HOTAIR	Non-small cell lung cancer	Cisplatin	HOTAIR contributes to cisplatin resistance via downregulation of P21	[58]
SCAL1	Non-small cell lung cancer	Gefitinib	SCAL1 is overexpressed in lung cancer cells with elevated expression of NrF2	[53, 54]

#### Alterations in drug targets by lncRNAs

There is accumulating evidence indicating that there is heterogeneity among various carcinoma cells, such as pancreatic [59], breast [60], and prostate [61]. Cancer stem cells (CSCs) have the capacity for self-renewal and they differentiate to produce cancer cells [62]. Cancer cells derived from CSC have genes that when expressed, induce epithelial-mesenchymal transition (EMT), which plays an important role in mediating metastasis [62, 63]. Emerging evidence suggests that lncRNAs mediate tumorigenesis and drug resistance in certain types of cancers. Below, we will discuss the potential of lncRNAs as novel therapeutic targets for chemoresistance and targeted drug therapy to prevent and treatment drug-resistant cancers.

Gefitinib is a tyrosine kinase inhibitor (TKI) that antagonizes the epidermal growth factor receptor (EGFR) [64]. However, it has been previously reported that cancer cells can develop resistance to gefitinib during treatment by the following mechanisms: a second mutation in the EGFR protein [65], c-MET amplification [66], changes in signaling pathways, such asIL-6/JAK1/STAT3 [67], PIK3CA [68] and IGF1R [69], activating mutations, RAS-MAPK pathway activation [70], and by alterations in the tumor microenvironment [71]. A recent study reported that gefitinib resistance in NSCLC is mediated by the overexpression of LINC00665 and that the loss of LINC00665 reduces the activation of the EGFR and Akt pathways (which decreases cell proliferation and survival) by interacting with the enhancer of the zeste 2 polycomb repressive complex 2 subunit (EZH2) [72].

Specific drug targets of lncRNAs have been reported to affect the progression stages in prostate cancer (PCa) [73]. In addition, since the emergence of next-generation sequencing, there is evidence indicating that PCa is a molecularly heterogeneous cancer [61]. The most salient therapeutic target in PCa is the androgen receptor (AR) [74]. In hormone-sensitive PCa, AR signaling is regulated by lncRNAs, such as HOTAIR, which represses the degradation of the E3-ubiquintin - AR complex, which induces castration resistant prostate cancer (CRPC) to promote metastasis of cancer cells [75]. Gu et al. [73] reported that the lncRNA bladder and prostate cancer suppressor (LBCS) protein is overexpressed CRPC cells and tissues, which inhibits PCa growth under castration conditions by blocking AR signaling [73]. In hormone sensitive PCa, transforming growth factor-beta (TGF-beta) activates the expression of the long noncoding RNA activated by TGF-beta (lncRNA-ATB), which upregulates the levels of certain EMT molecules in CRPC and increases cyclin D1 and cyclin E levels which increase cell proliferation and EMT [76]. In contrast, the differentiation antagonizing non-protein coding RNA (DANCR) produces an accelerated terminal differentiation of normal prostate epithelial cells and reverses AR signaling, repressing the metastasis in PCa cells [77, 78]. Thus, it is possible that PCa metastasis may be mediated by positive or negative lncRNAs, depending on the subtype of hormone-sensitive receptors. Furthermore, the lncRNA AR splice variant 7 and AS region of C-terminal binding protein 1 (CTBP1-AS) downregulates CTBP1 expression by recruiting the RNA-binding transcriptional repressor, PSF, and together with histone deacetylases, accelerates progression to CRPC in PCa cells [79, 80]. Recently, Ta et al. [81] reported a significant positive correlation between the expression of the novel hormone-upregulated lncRNA within LCK (HULLK) and resistance to AR signaling. HULLK is encoded within the lymphocyte-specific protein tyrosine kinase (LCK), which is regulated by androgen receptors [81]. In the presence of AR, the loss of HULLK significantly decreased cancer cells proliferation, whereas the overexpression of HULLK increased the sensitivity of PCa cells to AR in CRPC [81]. Overall, these data may be useful in finding novel biomarkers or more effective therapeutic targets for clinically resistant PCa.

#### The effect of lncRNAs on EMT in the cytoplasm and the nucleus

**Fig. 3:**
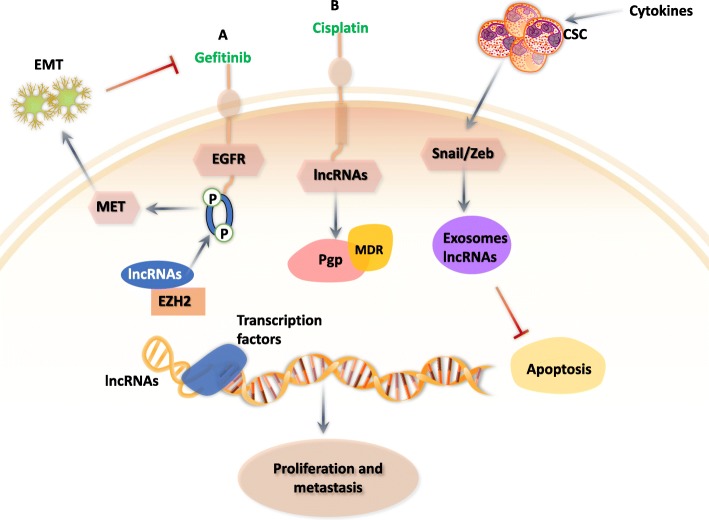
Schematic illustration of lncRNA-induced resistance to anticancer drugs by altering drug targets and EMT progression

The EMT plays an important role in cancer progression, metastasis and drug resistance [59, 82]. In various cancers, EMT is defined as the transformation from epithelial cells to a mesenchymal phenotype [82] (Fig. 3).

Recent studies have shown a significant positive correlation between the expression of EMT and CSCs and an increase in cancer cell metastasis and resistance [83]. In aggressive cancer cells, the expression of EMT markers increased the stemness of tumor-initiating CSCs and the magnitude of invasiveness and metastasis [59].

LncRNAs mediate pathophysiological processes associated with hepatocellular carcinomas and regulate invasiveness and drug resistance. The lncRNA miR503HG is minimally expressed in HCC and when expressed at high levels, it inhibits HCC metastasis by regulating the heterogeneous nuclear ribonucleoprotein A2/B1 (HNRNPA2B1)/NF-κB signaling pathway [84].

In squamous cell carcinomas (SCCs), such as esophagus, head and neck, lung and skin cancers, lncRNA have “super-enhancer” regions that are involved in inducing the expression of certain genes. For example, the transcription factors, tumor protein p63 (TP63) and SRY-box transcription factor 2 (SOX2), which co-bind to the promoter and super-enhancer regions of the lncRNA CCAT1, regulate lineage-specific expression patterns in esophageal SCC [85]. The TP63/SOX2/CCAT1 complex activates EGFR, which activates its downstream signaling pathways in esophageal SCC [85]. MALAT1 is a classical lncRNA that mediates alternative splicing, metastasis and recurrence in several types of cancers [86]. Recent evidence suggests that MALAT1 interacts with c-MYC, and this complex binds to the Kinectin 1 promoter region to enhance EGFR protein expression in SCC [87]. The MALAT-KTN1-EGFR axis plays a pivotal role in SCC progression [87].

LncRNAs, such as epidermal growth factor-like domain-containing protein 7 (EGFL7), which increases cancer cell proliferation and metastasis, have been identified and validated as clinical targets for genitourinary cancers [88]. Zhai et al. [88] have shown that the novel lncRNA URRCC accelerates the progression of renal cell carcinoma (RCC). The expression of high levels of URRCC are positively correlated with increased tumor volume, clinical stages and overall survival of patients [88]. URRCC binds to the EGFL7 promoter, resulting in the acetylation of EGFL7 at the histone H3 residue, increasing Akt signaling, while inhibiting FOXO3 signaling, facilitating RCC proliferation and invasion in RCC [88].

#### EMT and exosomal lncRNA in cancer

Exosomes are 30–100 nm in diameter, composed of proteins, RNA, and DNA [89], and are secreted by extracellular vesicles and released by exocytosis [90]. Exosomal noncoding RNAs have been found in the blood, urine, breast milk, saliva, and various tissues [91]. There is increasing evidence indicating that exosomal RNAs are present in various cancers and they enhance cancer progression, invasion, metastasis and tumorigenesis [92]. The levels of lncRNAs can be either increased or decreased in exosomes from cancer-associated fibroblasts (CAFs), which facilitate the transition of cancer cells to the EMT [90] (Fig. 3).

The metastasis of cancer cells can be affected by exosomal lncRNAs. For example, the exosomal FMR1 antisense RNA1, FMR1-AS1, in female esophageal squamous cell carcinoma (ESCC), maintains ESCC and CSC dynamic interconversion, by activating the TLR7/NFκB/c-Myc signaling pathway in ESCC [93]. Gastric cancer cells produce and secrete the exosomal HOXA distal transcript antisense RNA (HOTTIP) and it is superior to traditional biomarkers in the serum of cancer patients, such as cancer embryonic antigen (CEA), cancer antigen 19–9 and cancer antigen 72–4 [94]. The lymph node metastasis-associated transcript 2 (LNMAT2) increases lymphatic metastasis in bladder cancer, most likely by binding to the prospero homeobox 1(PROX1) promoter, where it regulates PROX1 transcription by inducing hnRNPA2B1-mediated H3 lysine 4 trimethylation, resulting in lymphangiogenesis and lymphatic metastasis [95]. Exosomes can also be affected by lncRNAs. Indeed, the lncRNA activated by the anaphase-promoting complex subunit 1 (APC1) regulator of the Wnt signaling pathway (lncRNA-APC1), which has a tumor-suppressive role in colorectal carcinoma (CRC), is activated by peroxisome proliferator-activated receptor alpha (PPAR-α), which decreases the production of exosomes [96] (Table 2).

**Table 2:** Drug targets and EMT related lncRNAs in anti-cancer drug resistance

lncRNAs	Up/down	Targets	Mechanisms and function	Cancers	Refs
lncRNA LBCS	up	AR	AR activation	PCa	[73]
HOXC-AS3	up	H3K4me3 and H3K27	By YBX1 regulating, promotes H3K4me3 and H3K27 acetylation	GC	[97]
miR503HG	Down			HCC	[84]
HOTAIR	up			PCa	[75]
CCAT1	up	EGFR	TP63 and SOX2 co-bind to the promoter and super-enhancer regions of CCAT1	SCC	[85]
URRCC	up	EGFL7/P-AKT/FOXO3	AKT signaling pathway Proliferation and metastasis	RCC	[88]
lncRNA GUARDIN	up	TRF2	p53-responsive lncRNA	Various cancers	[98]
Linc00210	up	CTNNBIP1	Wnt/β-catenin signaling activation	liver cancer	[99]
Linc00659	up	cycle-related genes		colorectal cancer	[100]
LINC01133	up	APC	Wnt/β-catenin pathway	gastric cancer	[101]

#### Epigenetics and related lncRNAs

**Fig. 4:**
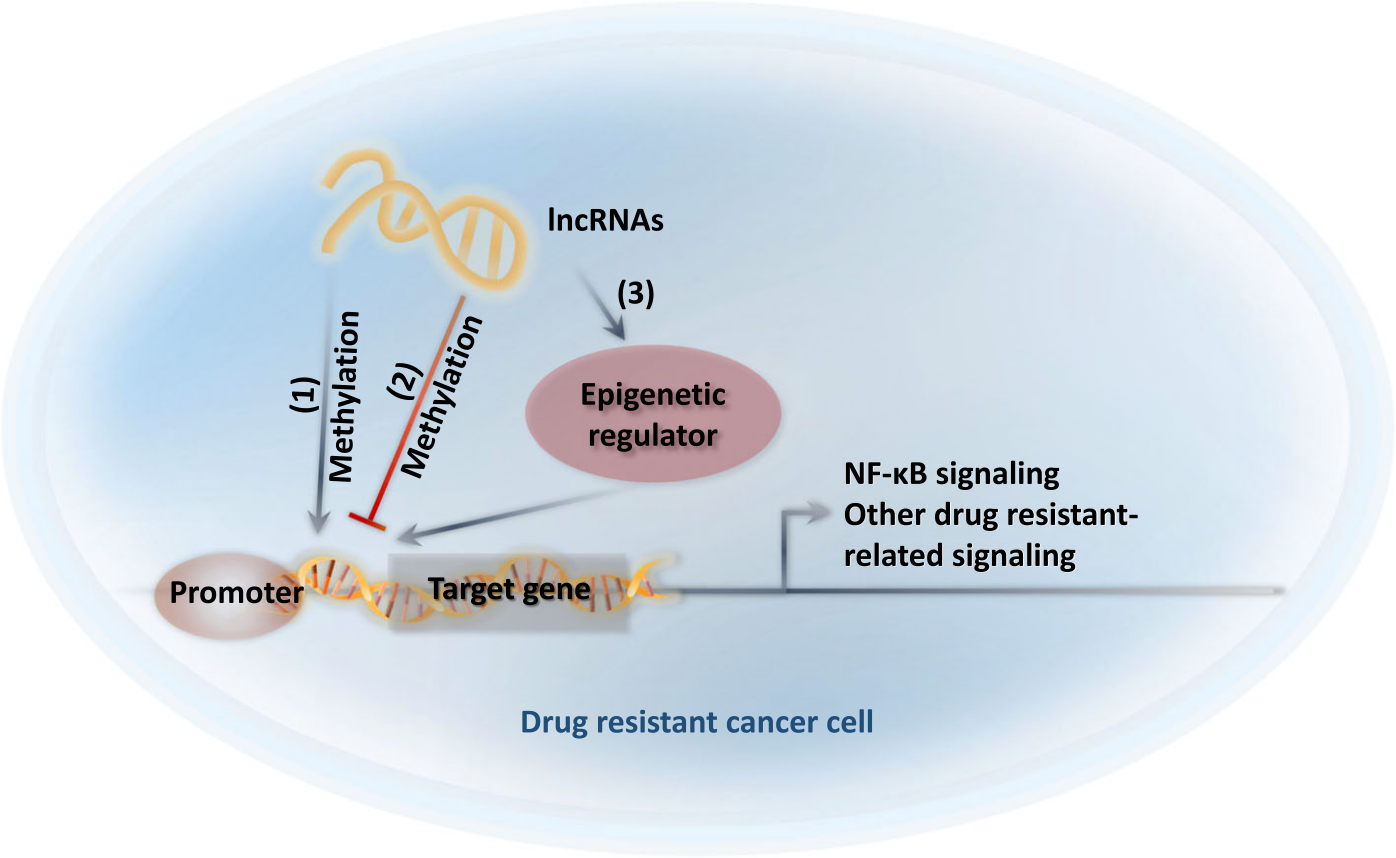
Schematic illustration of the effect of lncRNAs on gene expression

It has recently been postulated that drug resistance is linked to genetic factors (drug – induced mutations) and epigenetic factors (drug - induced non-mutational alterations of gene function) [5, 102]. LncRNAs can regulate epigenetic modifications involved in cell cycle, cell differentiation, development, and pluripotency of cancers [103, 104]. Their involvement in epigenetic processes include the recruitment of histone-modifying enzymes and DNA methyltransferases, leading to the establishment of chromatin conformation patterns that result in the specific regulation of certain genes [105]. In the material below, we discuss cancer-related lncRNAs that regulate epigenetic changes by histone modifications, DNA methylation and chromatin architecture (Table 3) (Fig. 4).

**Table 3:** LncRNAs with an epigenetic function in cancer

Name	Cancer	Mechanism	Ref.
*ecCEBPA*	Upregulated in gastric cancer; inverse correlation with CEBPA in leukaemia cell lines.	association with DNMT1 regulates DNA methylation	[106–108]
Xist	Abnormal expression in hematologic cancer.	① influences X reactivation and results in genome-wide changes; ②directly interacts with SHARP to silence transcription through HDAC3; ③binds PRC2(the epigenetic complex responsible for trimethylation of histone H3 lysine 27 methylation), and targets PRC2 to Xi;	[109–111]
HOTAIR	Upregulated in epithelial cancer cells, such as primary breast tumors and metastases, gastric cancer, oral squamous cell carcinoma glioblastoma multiforme, colorectal cancer, esophageal squamous cell carcinoma etc., and promotes cancer metastasis.	① Induces genome-wide re-targeting of PRC2 to an occupancy pattern, leading to altered histone H3 lysine 27 methylation, and increased cancer invasiveness and metastasis in a manner dependent on PRC2. ②HOTAIR promotes EMT by switching histone H3 lysine 27 acetylation to methylation at the E cadherin promoter, which induces the transcriptional inhibition of E cadherin. ③interacts with PRC2 and LSD1 complex, and as a histone scaffold, to inhibit the transcription of the HOXD cluster	[112–117]
H19	Upregulated in different cancer types, such as colorectal cancer, breast cancer, ovarian cancer cells, etc., and promotes oncogenesis and drug resistance.	① Interacts with SAHH to regulate the DNMT3B - dependent DNA methylation at different genetic loci. ② The impact of H19 on metastasis could be due to the sequestration of different microRNAs	[12, 118–121]
MITA1	A new identified energy stress-inducible lncRNA that promotes hepatocellular carcinoma metastasis	MITA1 may regulate the transcription of Slug to promote the epithelial-mesenchymal transition	[122]
TARID	Deregulated in various human cancers	Recruits the DNA demethylation regulator, GADD45α, to activate the transcription of the tumor suppressor gene, TCF21. GADD45A is an epigenetic R-loop reader that recruits the demethylation machinery to promoter CGIs.	[123, 124]
MALAT1	Upregulated in lung cancer, gastric cancer, colorectal cancer, hepatocellular carcinoma, thoracic aortic aneurysm; Deregulated in breast cancer	① Oct4 transcriptionally activates MALAT1 via enhancer binding to promote cell proliferation and motility, causing lung tumorigenesis and poor prognosis. ② MALAT1 acts as a competing endogenous RNA for miR-23b-3p and attenuates the inhibitory effect of miR-23b-3p in GC cells. ③ the rs664589 G allele alters the binding of MALAT1 to miR-194-5p, resulting in increased expression of MALAT1 in colorectal cancer; ④ MALAT1 regulates cancer glucose metabolism, enhancing glycolysis, and inhibiting gluconeogenesis via elevated translation of the transcription factor TCF7L2. ⑤ MALAT1 binds and inactivates the prometastatic transcription factor TEAD, preventing TEAD from associating with its co-activator, YAP, and target gene promoters in breast cancer. ⑥ interacts with DBC1 to regulate p53 acetylation. ⑦ The HDAC9-MALAT1-BRG1 complex binds chromatin and represses contractile protein gene expression in association with gain of histone H3-lysine 27 trimethylation modifications.	[125–131]
NEAT1	Upregulated in lung cancer	Oct4 transcriptionally activates NEAT1 via promoter binding to facilitate cell proliferation and motility, causing lung tumorigenesis and poor prognosis.	[126]
ANRIL	High expression linked to poor outcome. ANRIL was identified as an oncogene in a number of tumors such as acute myeloid leukemia, gastric cancer, lung cancer, hepatocellular carcinoma, and esophageal squamous cell carcinoma.	① represses the expression of adiponectin receptor (AdipoR1), which is a key regulator of glucose metabolism, which affects the phosphorylation of AMPK and SIRT1. ② represses KLF2 transcription by binding to PRC2 and recruiting it to the KLF2 promoter region.	[132–135]
AFAP1-AS1	High expression linked to poor outcome in non-small cell lung cancer	AFAP1-AS1 interacts with EZH2 and recruits EZH2 to the promoter regions of p21, epigenetically repressing p21 expression.	[136]

The lncRNA HOTAIR plays a role in cancer metastasis and its levels are increased in epithelial cancer cells, such as breast cancer [112], gastric cancer [113], oral squamous cell carcinoma [114], glioblastoma multiforme [115], colorectal cancer [116], and esophageal squamous cell carcinoma [117]. LncRNA H19 expression is increased in various types of cancers and it regulates DNA methylation genome wide by regulating S-adenosylhomocysteine hydrolase to promote oncogenesis and drug resistance [118]. The lncRNA extra coding CCAAT enhancer binding protein alpha (ecCEBPA) is upregulated in gastric cancer cells [106]. The lncRNA ecCEBPA regulates DNA methylation at the CEBPA gene locus due to the interaction of ecCEBPA with DNA methyltransferase 1(DNMT1) [107, 108]. The lncRNAs Xist and HOTAIR interact with the proteins polycomb repressive complex 2 (PRC2) and lysine-specific demethylase 1 (LSD1) to prevent the transcription of target genes, such as HDAC3 and E-cadherin, and regulate cancer metastasis [109, 112, 113].

## Conclusions and perspectives

**Fig. 5:**
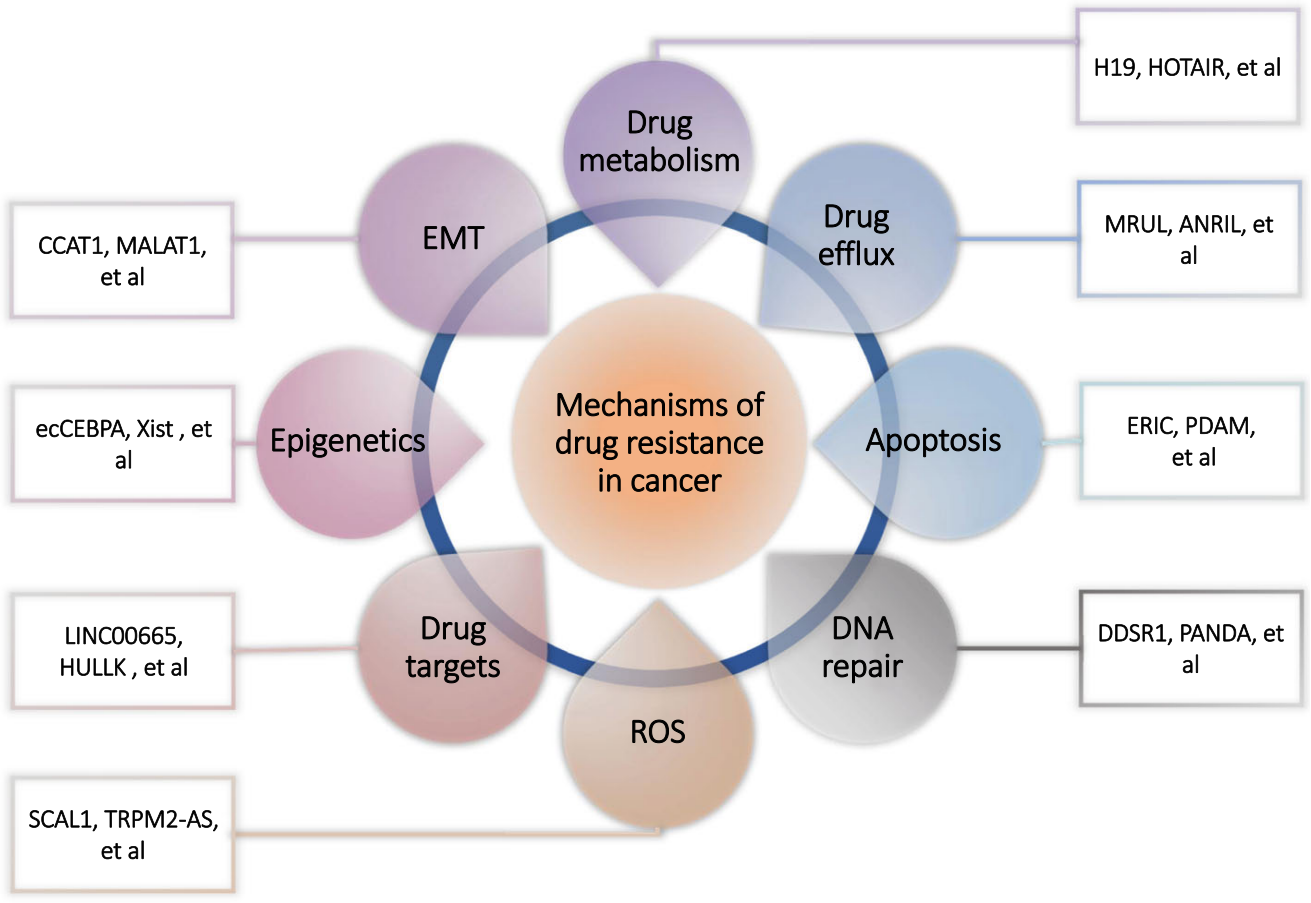
Schematic illustration of the effect of lncRNAs on drug resistant cancer cells

Apart from altered drug metabolism, drug efflux, DNA damage repair, ROS, cell death, drug target, EMT, epigenetic factors, autophagy, oncogenes and microRNAs, lncRNAs have been shown to produce drug resistance in certain types of cancer cells. LncRNAs are also involved in many cellular and genomic process and recent research indicates their involvement in carcinogenesis. Currently, different lncRNAs have been shown to induce chemoresistance in cancer cells (Fig. 5). Further research is required to identify additional lncRNAs that may be associated with cancer cell drug resistance and delineate their roles in carcinogenesis and chemoresistance. Overall, accumulating research indicates that targeting lncRNAs may be a strategy for the treatment of drug resistance in cancer cells.

## Data Availability

Not applicable.

## Abbreviations

5-FU: 5-fluorouracil
ABC: ATP bind cassette
AdipoR1: Adiponectin receptor
ALDH: Aldehyde dehydrogenase
ANRIL: Antisense non-coding RNA in the INK4 locus
APC1: Anaphase-promoting complex subunit 1
AR: Androgen receptor
BLACAT1: Bladder cancer associated transcript-1
CAFs: Cancer-associated fibroblasts
CEA: Cancer embryonic antigen
CHST15: Carbohydrate sulfotransferase
CRC: Colorectal carcinoma
CRPC: Castration resistant prostate cancer
CSCs: Cancer stem cells
CTBP1-AS: C-terminal binding protein 1
CUDR: Cancer upregulated drug resistant gene
DANCR: Differentiation antagonizing non-protein coding RNA
DDSR1: DNA damage-sensitive RNA1
DNMT1: DNA methyltransferase 1
Dox/ADR: Doxorubicin/adriamycin
ecCEBPA: Extra coding CCAAT enhancer binding protein alpha
EGFL7: Epidermal growth factor-like domain-containing protein 7
EGFR: Epidermal growth factor receptor
EMT: Epithelial-mesenchymal transition
ERIC: E2F1-regulated inhibitor of cell death
ESCC: Esophageal squamous cell carcinoma
EZH2: Enhancer of the zeste 2 polycomb repressive complex 2 subunit
GSTM3TV2: Glutathione S-transferase mu 3, transcript variant 2 and noncoding RNA
HCC: Hepatocellular cancer
HIF-1α: Hypoxia-inducible factor alpha
HNRNPA2B1: Heterogeneous nuclear ribonucleoprotein A2/B1
HOTAIR: HOX transcript antisense intergenic RNA
HOTTIP: HOXA distal transcript antisense RNA
HULLK: Hormone-upregulated lncRNA within LCK
LAT2: L-type amino acid transporter 2
LBCS: lncRNA bladder and prostate cancer suppressor
LCK: Lymphocyte-specific protein tyrosine kinase
lncRNA-ATB: Long noncoding RNA activated by TGF-Beta
lncRNAs: Long non-coding RNAs
LNMAT2: Lymph node metastasis-associated transcript 2
LSD1: Lysine-specific demethylase 1
MALAT1: Metastasis-associated lung adenocarcinoma transcript 1
MDR: Multidrug resistance
MRUL: MDR-related and upregulated lncRNA
mTOR: Mammalian target of rapamycin
NFYA: Nuclear transcription factor Y subunit alpha
Nrf2: Nuclear factor erythroid 2-related factor 2
NSCLC: Non-small cell lung cancer
OLR1: Oxidized low-density lipoprotein receptor 1
PANDA: p21 associated ncRNA DNA damage activated
PCa: Prostate cancer
PCGEM1: Prostate cancer gene expression marker 1
PDAM: p53-dependent apoptosis modulator
P-gp: p-glycoprotein
PPAR-α: Peroxisome proliferator-activated receptor alpha
PRC2: Polycomb repressive complex 2
PROX1: Prospero homeobox 1
PVT1: Plasmacytoma variant translocation 1
RCC: Renal cell carcinoma
ROS: Reactive oxygen species
SCAL1: Smoke and cancer-associated lncRNA-1
SCCs: Squamous cell carcinomas
SGK1: Serum and glucocorticoid-inducible kinase 1
SOX2: SRY-box transcription factor 2
TGF-beta: Transforming growth factor-beta
TKI: Tyrosine kinase inhibitor
TP63: Tumor protein p63
TRPM2-AS: Transient receptor potential cation channel subfamily M member 2 antisense
UCA1: Urothelial cancer associated 1
VCR: Vincristine
VLDLR: Very low density lipoprotein receptor
XIST: X-inactive specific transcript
